# Targeting Glutaminase Isoforms GLS and GLS2 in Luminal Breast Cancer

**DOI:** 10.3390/ijms27062780

**Published:** 2026-03-19

**Authors:** Brendah K. Masisi, Rokaya El Ansari, Ali Fakroun, Büsra Erkan, Emad A. Rakha, Andrew R. Green

**Affiliations:** 1 Nottingham Breast Cancer Research Centre, Academic Unit for Translational Medical Sciences, School of Medicine, University of Nottingham Biodiscovery Institute, University Park, Nottingham NG7 2RD, UKrokayaelansari@gmail.com (R.E.A.); mzxbe2@exmail.nottingham.ac.uk (B.E.);; 2 School of Allied Health Professions, Faculty of Health Sciences, University of Botswana, Plot 4775 Notwane and Mobuto Road, Gaborone P/Bag UB 0022, Botswana; 3 Department of Cellular Pathology, Nottingham University Hospitals NHS Trust, Nottingham City Hospital, Hucknall Road, Nottingham NG5 1PB, UK

**Keywords:** breast cancer, luminal cell lines, glutaminase, metabolism

## Abstract

Upregulation of glutaminase enzymatic activity promotes tumour cell proliferation. Its overexpression correlates with poor disease outcome in patients, including those with breast cancer. A selective glutaminase inhibitor, CB-839, which targets cancer cells by blocking glutamine conversion to glutamate, has shown promising preclinical results as a therapeutic target in triple-negative breast cancer treatment. The current study aimed to determine the importance of glutaminase in Oestrogen Receptor positive/luminal breast cancer to potentially identify therapeutic targets to treat this subtype. In vitro studies using luminal breast cancer cells were performed to investigate the effects of siRNA knockdown of glutaminase genes (*GLS* and *GLS2*) and inhibition using CB-839 on functional assays. Silencing *GLS* in luminal breast cancer cells significantly reduced cell proliferation whilst inducing apoptosis. A similar impact on cell proliferation was observed when silencing *GLS2* in luminal B cells, but there was no observed effect on cell apoptosis and cell cycle. There was little effect of GLS inhibition using CB-839 in luminal breast cancer. This study demonstrates that glutaminase is necessary for luminal breast cancer growth and survival. Co-targeting *GLS* and *GLS2* might be a novel approach for the treatment of this subclass. Further functional studies to evaluate the underlying molecular mechanisms of this process are warranted.

## 1. Introduction

Therapeutic strategies for breast cancer (BC) patients are guided by the expression status of biomarkers: Oestrogen Receptor (ER), Progesterone Receptor (PR), and Human Epidermal Growth Factor Receptor 2 (HER2). ER+ve or luminal tumours account for approximately 70% of all BC [1]. Molecular profiling has provided a biological classification system for categorising luminal BC into two molecular subtypes, namely luminal A and the more aggressive luminal B. By using immunohistochemistry, these subtypes have been confirmed and classified as follows: luminal A (ER+ve, PR+ve, HER2-ve, KI67 < 10%) and luminal B (ER+ve, PR+ve, HER2+ve or HER2-ve, KI67 ≥ 10%) [2]. Endocrine therapies, such as using tamoxifen, which is a selective ER modulator, or aromatase inhibitors, which cause oestrogen depletion, remain the key drugs for the treatment of luminal BC in postmenopausal women, particularly in the context of post-surgical adjuvant therapy [3]. Evidence shows that treatment with endocrine therapy inhibits the action of oestrogen, significantly improving survival outcomes and reducing recurrence in patients with ER+ve BC [4]. Patients presenting with aggressive luminal BC can relapse or be resistant or acquire resistance to contemporary treatments, where the risk of tumour recurrence and relapse persists beyond five years of treatment and remains a clinical and scientific challenge [5]. Among ER+ve BC, aggressive luminal B tumours are associated with a significantly worse prognosis compared with the luminal A subtype, and a subset of patients may develop resistance to therapy and ultimately experience disease-related death [4,6].

Deregulation of cellular energetics is one of the components of the hallmarks of cancer. Glutamine is a crucial amino acid that has a role in providing energy for normal and proliferating cells/tissues. Cancer cells relying on glutaminolysis show an increased activity of glutaminase in proliferating tumours compared with their normal counterparts [7,8]. There are two glutaminase forms, GLS and GLS2, which are encoded by the genes *GLS* and *GLS2*, located on distinct chromosomes, and can also exist as different splice variants [9,10]. The ‘kidney-type’ GLS is the main isoform and is highly upregulated in most cancers, resulting in increased rates of glutamine metabolism [9,11,12]. Glutamine metabolism begins with glutamine being catalysed to glutamate by GLS, which is converted to α-ketoglutarate and is further processed within the TCA cycle for further use in biological processes by cells [13].

Evidence shows that GLS is upregulated in highly proliferative BC subtypes. El-Ansari et al. have previously shown that glutaminase plays a vital role in aggressive luminal BC [14]. A positive correlation between GLS and glutamine metabolism-related markers has been established, which suggests that GLS is important in BC biology [14,15]. On the other hand, GLS2 shows tumour-suppressive activity, where GLS2 is primarily associated with well-differentiated tumours [16,17]. In BC, GLS2 levels are higher in ER+ve/luminal tumours relative to normal tissue [18]. In brain tumours, GLS2 is a p53 target, reducing tumour growth and colony formation by mediating p53 in the cellular regulation of antioxidant defence [19].

The role and importance of glutaminase in aggressive luminal BC remain unclear and contradictory. Preclinical studies have shown antitumour activity through both GLS enzyme inhibition and *GLS* mRNA knockdown, supporting this approach as a potential strategy to suppress autonomous cancer growth across multiple tumour types, which include triple-negative BC, non–small cell lung cancer (NSCLC), melanoma, and renal cell carcinoma (RCC) [18,20,21].

There are limited effective treatments for patients with aggressive luminal BC; thus, blocking the activity of GLS/GLS2 or silencing the genes could reduce BC cell proliferation and be a promising therapeutic target. This study, therefore, aims to decipher the role of GLS and GLS2 in luminal BC using in vitro models to assess the effects of knockdown and inhibition on cell proliferation, apoptosis, glutamine uptake, and glutaminase activity.

## 2. Results

### 2.1. Effect of GLS and GLS2 Knockdown in Luminal BC

**Figure 1:**
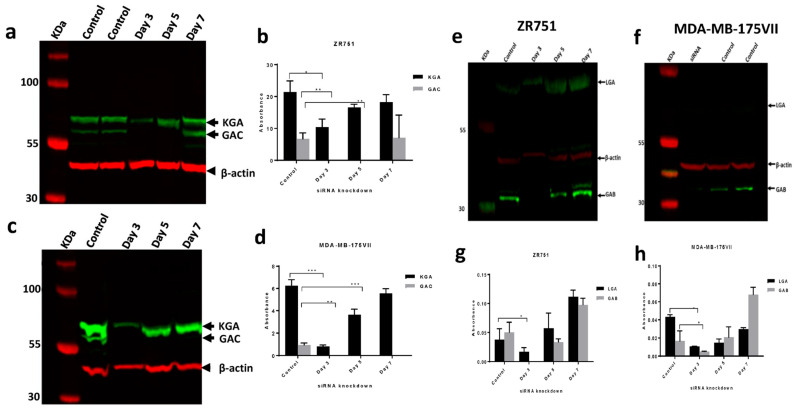
Glutaminase knockdown in luminal B breast cancer cell lines transfected with siRNA designed against *GLS* and *GLS2* and control cells. Western blot images for GLS expression in (**a**) ZR-75-1 and (**c**) MDA-MB-175VII cells transfected with *GLS* siRNA, along with negative scrambled siRNA. Bar graphs (**b**,**d**) show GLS protein expression at days 3, 5, and 7 post-transfection. Bands were observed at the predicted size: 73 kDa and 65 kDa, relating to the predicted size of the KGA/GAC GLS isoforms. Western blot images for GLS2 post-transfections in ZR-75-1 and MDA-MB-175VII. Western blot images (**e**,**f**) show that *GLS2* was efficiently silenced in ZR-75-1 and MDA-MB-175VII cells, respectively. Bands at the predicted size of 65 kDa and 31 kDa relating to the predicted size of the GLS2 isoforms LGA and GAB were observed. Bar graphs (**g**,**h**) show quantification of GLS2 protein at days 3, 5, and 7 post-transfection. β-actin was used as a loading control. Statistical significance was calculated with unpaired *t*-tests. Data displayed as mean ± SEM, * *p* < 0.05, ** *p* < 0.01, *** *p* < 0.001.

**Figure 2:**
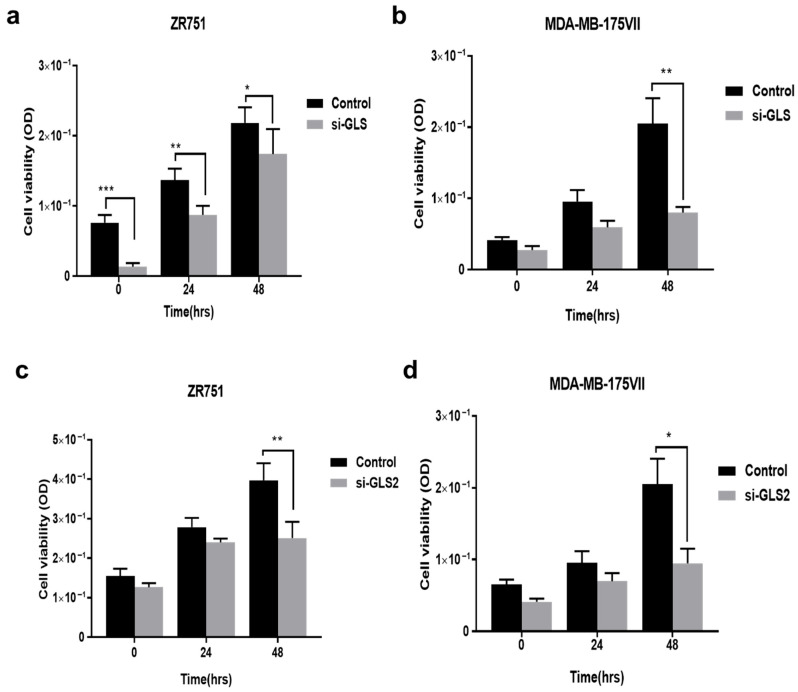
The effect on cell proliferation in luminal B breast cancer cells upon *GLS* or *GLS2* knockdown: (**a**,**c**) ZR-75-1 and (**b**,**d**) MDA-MB-175VII compared to controls using MTS viability assay. Figures are representative of three independent experiments. Statistical significance was calculated with unpaired *t*-tests. Data displayed as mean ± SEM, * *p* < 0.05, ** *p* < 0.01, *** *p* < 0.001.

*GLS* was efficiently silenced in both luminal BC cell lines at 72 h of transfection, as shown by the loss of GLS protein in Western blots compared with the controls (Figure 1a,c). Protein expression of GLS2 was efficiently silenced by si-*GLS2* in ZR-75-1 and MDA-MB-175VII transfected cells at 72 h post-transfection (Figure 1e,f). When comparing knockdown cells with the controls, knockdown of *GLS* and *GLS2* resulted in decreased cell proliferation in ZR-75-1 and MDA-MB-175VII (*p* < 0.01, *p* = 0.006, respectively; Figure 2). Cell proliferation decreased in ZR-75-1 at 24 h and 48 h, whereas in MDA-MB-175VII the same was observed only at 48 h. Similarly, cell proliferation was decreased in both cell lines transfected with *GLS2* at 48 h (*p* = 0.009, *p* = 0.02, respectively, Figure 2).

**Figure 3:**
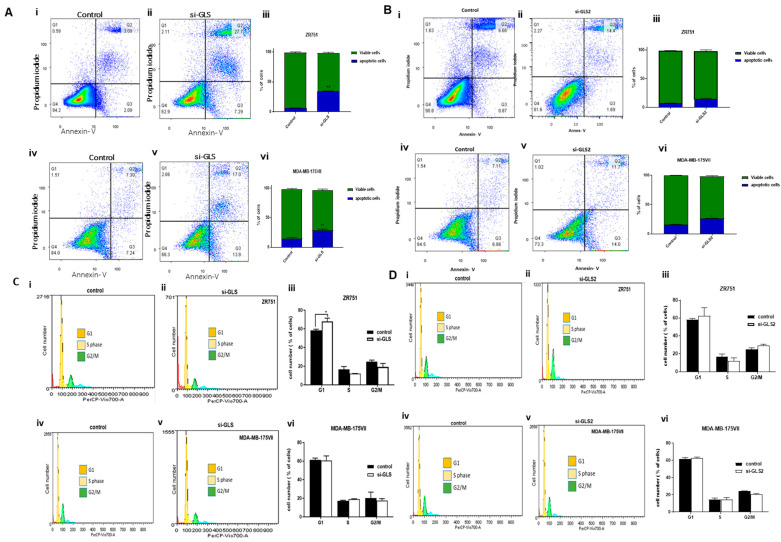
Effect of *GLS* and *GLS2* knockdown on apoptosis and cell-cycle distribution in siRNA-transfected ZR-75-1 and MB-MDA-175VII cells using flow cytometry. Figures (**A**,**B**) (**i**,**ii**,**iv**,**v**) are representative apoptosis assays for ZR-75-1 and MB-MDA-175VII and GLS and GLS2 knockdown cells, respectively. Bar graphs (**A**,**B**) (**iii**,**vi**) represent the percentage of apoptotic cells following GLS and GLS2 knockdown in ZR-75-1 and MB-MBA-175VII, respectively. Figures (**C**,**D**) (**i**,**ii**,**iv**,**v**) are representative cell-cycle assays for ZR-75-1 and MB-MDA-175VII and GLS and GLS2 knockdown cells, respectively. Bar graphs (**C**,**D**) (**iii**,**vi**) represent the cell- cycle analysis following GLS and GLS2 knockdown in ZR-75-1 and MB-MBA-175VII, respectively. Statistical significance was calculated with unpaired *t*-tests. Data represent three independent experiments and are shown as mean ± SEM; * *p* < 0.05, ** *p* < 0.01.

**Figure 4:**
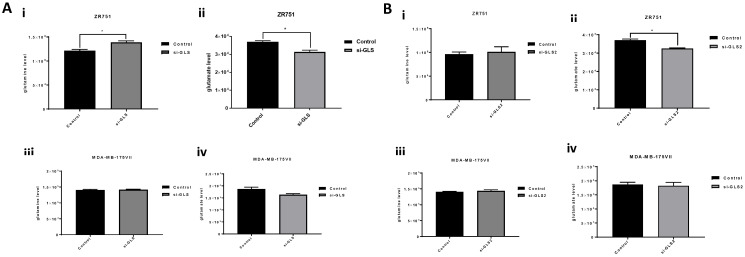
Glutamine and glutamate levels following *GLS* and *GLS2* knockdown. Bar graphs illustrate the impact of knockdown in (**A**,**B**) (**i**,**ii**) ZR-75-1 cells and (**A**,**B**) (**iii**,**iv**) MDA-MB-175VII cells, compared with control cells, as measured using the glutamine/glutamate assay; * *p* < 0.05.

Knockdown of *GLS* induced apoptosis in both ZR-75-1 and MB-MDA-175VII with an increase of 34% (*p* = 0.003) and 29% (*p* = 0.008), respectively (Figure 3A(i–vi)). Moreover, knockdown of *GLS* induced early (Q3) and late apoptosis (Q2) in both ZR-75-1 (7%, 28%) and MB-MDA-175VII (14%, 17%) compared to control cells (Figure 3A(i–vi)). MDA-MB-175VII cells transfected with *GLS2* siRNA saw an increase in apoptosis (24%) relative to the transfected control cells (*p* = 0.03; Figure 3B(vi)). The percentage of ZR-75-1 cells in G_1_ phase was significantly increased when *GLS* expression was knocked down compared to untransfected control cells (*p* = 0.04; Figure 3C(i–iii)). There was no change in S or G_2_/M phases. No changes were observed in cell-cycle distribution in MDA-MB-175VII following *GLS* knockdown (Figure 3C(iv–vi)). Furthermore, *GLS2* knockdown had no measurable effect on the cell cycle in either ZR-75-1 or MDA-MB-175VII (Figure 3D). Knockdown of *GLS* slightly increased glutamine by 5% (*p* = 0.045) and decreased glutamate (*p* = 0.038) levels by 70% in ZR-75-1 (Figure 4A(i,ii)). For MDA-MB-175VII, no difference in the levels of glutamine or glutamate upon *GLS* knockdown compared to control cells was observed (Figure 4A(iii,iv)). There was no effect on glutamine levels when *GLS2* was knocked down compared with the control. However, *GLS2* knockdown showed a decrease in glutamate levels in the ZR-75-1 (Figure 4B(ii), *p* = 0.02).

### 2.2. Effect of Glutaminase Inhibition in Luminal BC

**Figure 5:**
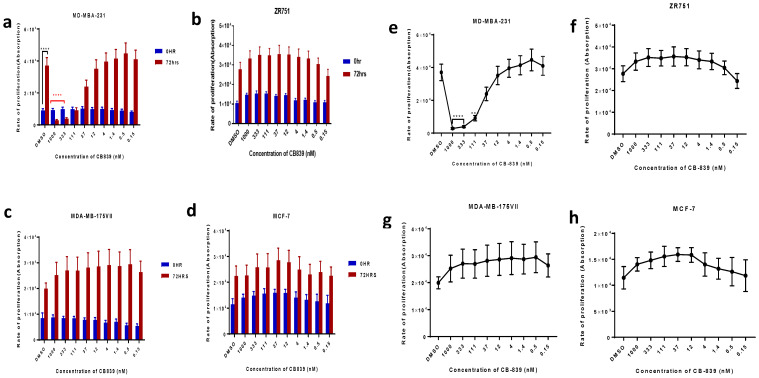
The effect of *GLS* inhibition on breast cancer cell proliferation in (**a**) MDA-MBA-231, (**b**) ZR-75-1, (**c**) MDA-MB-175VII and (**d**) MCF-7 breast cancer cell lines compared to vehicle control. Each cell line was exposed to concentrations of GLS inhibitor from 0.15 to 1000 nM and DMSO as a control. Graphs (**a**–**d**) show a comparison between each concentration and DMSO at 0 vs. 72 h. Graphs (**e**–**h**) shows rate of proliferation after 72 h of inhibition exposure only. Statistical significance was determined using one-way ANOVA and unpaired *t*-tests respectively. Data are presented as mean ± SEM from three independent experiments; *** *p* < 0.001, **** *p* < 0.0001.

There was no inhibitory effect on cell proliferation by CB-839 at any concentration in luminal BC cells: MCF-7, MDA-MB-175VII, or ZR-75-1 (Figure 5). Inhibition of GLS using CB-839 at the higher concentrations (333 and 1000 nm, respectively) significantly inhibited cell proliferation by 30% and 40% in triple-negative BC cells, MDA-MB-231 at 72 h (*p* < 0.0001; Figure 5a,e).

**Figure 6:**
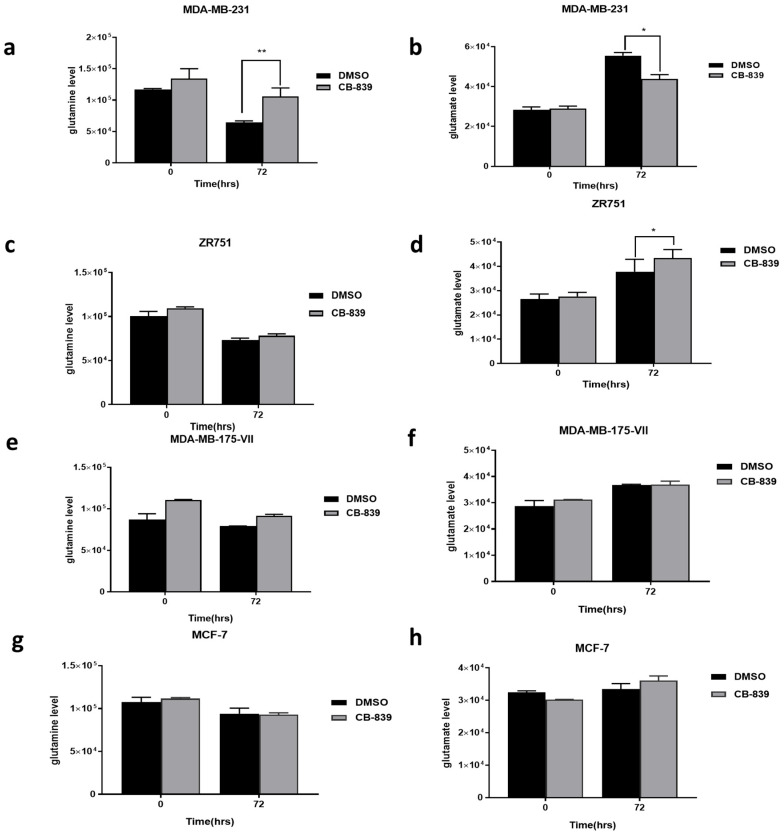
Effect of GLS inhibition on glutamine and glutamate production in (**a**,**b**) MDA-MB-231, (**c**,**d**) ZR-75-1, (**e**,**f**) MDA-MB-175VII, and (**g**,**h**) MCF-7 cells treated with 1000 nM CB-839 at 0 and 72 h. Data are presented as mean ± SEM from three independent experiments, and comparisons of treated cells with CB-839 and untreated cells were performed by unpaired *t*-test: * *p* < 0.05, ** *p* < 0.01.

Treatment with CB-839 for 72 h also significantly increased glutamine and reduced glutamate in triple-negative BC cells compared with the control by 40% and 20%, respectively (Figure 6a,b). There was a 10% increase in glutamate levels upon addition of GLS inhibitor after 72 h incubation in the luminal B ZR-75-1 cells (Figure 6d, *p* = 0.042). There was no significant effect of glutamine or glutamate observed in the remaining luminal cell lines: MDA-MB-175VII and MCF-7 (Figure 6e–h).

## 3. Discussion

Metabolic alterations in cancer cells have been an area of interest and rapidly growing research in the last two decades. Glutaminase isoenzymes have been a focus due to their association with metabolic reprogramming and their potential for cancer therapy. Although GLS is widely studied and documented, the function of GLS2 in BC has remained unclear and not well documented. Previous studies have provided evidence for GLS isoforms playing a role in the aggressive subclass of luminal BC in addition to triple-negative BC [14,22]. Interestingly, based on previous evidence, the two glutaminase isoforms have opposing roles in tumourigenesis [23]. Evidence has revealed that GLS2 expression occurs in malignant BC cells and in other cancer cells, such as colorectal, hepatoma, lymphocytic, and myeloid [19,21,24]. However, GLS2 variants are increased in well-differentiated tumours and have a tumour suppressive effect; thus, they result in improved survival of patients, whereas GLS expression in cancer is supportive of tumourigenesis [11]. GLS2 can be alternatively spliced to produce LGA and GAB variants [9,10]. Although the function of LGA, unlike GAB in tissues, is less well defined, there is evidence suggesting differential regulation of GLS2 transcripts in cancer [17]. Effects of GLS and GLS2 on cell-cycle distribution and apoptosis in luminal BC were explored. We demonstrated that *GLS* knockdown in ZR-75-1 inhibited cell proliferation as well as significantly arrested cells in the G_1_ phase in luminal BC cells. Apoptosis was also induced, indicating that disruption of cell-cycle progression is one mechanism by which GLS may act to inhibit cancer proliferation.

Similarly, MDA-MB-175VII cells exposed to anti-*GLS* siRNA decreased proliferation and induced apoptosis; however, there was no change in the proportion of cell-cycle phases. This observation could suggest that cancer cells may choose certain pathways that lead to immediate cell death without interfering with the cell cycle. These observations are consistent with former studies showing that blocking glutaminase activity causes cell-cycle arrest and apoptosis in some tumours [19,25]. Moreover, apoptosis, a form of programmed cell death, is characterised by several events that involve nuclear condensation, DNA fragmentation, cell shrinkage, and membrane disintegration. It is important to further understand the underlying mechanisms by which anti-apoptotic molecules exert their effects. Further protein validation of apoptosis using cleaved caspase 3 or PARP is warranted in the future, as it was not done in the current study.

To further elucidate the potential of inhibiting GLS in luminal B tumours, the selective GLS inhibitor CB-839 was utilised to assess the anti-proliferative effect on luminal BC cells. ZR-75-1 and MDA-MB-175VII were used in the study because they expressed the highest GLS protein levels based on quantification of the Western blot, and for comparison, luminal A cell lines were used. The current study used the TNBC cell line as a positive control because it has been previously shown to be sensitive to CB-839 [18]. As expected, CB-839 showed significant anti-proliferative effects in the triple-negative BC cells, MDA-MB-231, but did not display these in luminal B cells (ZR-75-1/MDA-MB-175VII) or luminal A cells (MCF-7 and T47D). It is clearly observed in the current study that knockdown of *GLS* significantly decreased proliferation. Our results showed that CB-839 did not display an inhibitory effect on proliferation in luminal BC, whereas in TNBC, there was a significant anti-proliferative effect. This finding may suggest a difference in the mechanism of inhibition of growth or hit off-targets in CB-839 and *GLS* siRNA.

The selective glutaminase inhibitor, CB-839, targets the activity of both GLS isozymes, but primarily the GAC splice variant, via the allosteric site [10]. In glutaminase-dependent triple-negative BC, evidence shows that treatment with CB-839 blocks glutamine utilisation, resulting in reduced proliferation and increased apoptosis [18]. In addition, CB-839 also decreases the mammalian target of rapamycin complex 1 (mTORC1) signalling pathway, which is regarded as the main regulator of cellular metabolism, promoting growth in response to the availability of nutrients and resulting in the cellular accumulation of glutamine and depletion of glutamate [26]. Altogether, these imply that GLS inhibition affects downstream glutaminolysis in triple-negative BC. In line with that, other studies have shown that knockdown of GLS in luminal cells did not affect cell proliferation, although they did not stratify between luminal A and luminal B tumours. Even though upon gene knockdown of *GLS*, cell proliferation was inhibited, the findings for treatment with CB-839 may suggest that luminal B cell lines are insensitive to GLS inhibition and are aligned with previous studies, which showed no sensitivity to CB-839.

This study proceeded to measure extracellular glutamine and glutamate in knockdown cells and CB-839-treated cells. Gross and colleagues have demonstrated that treatment of cells with CB-839 was capable of inhibiting GLS activation, raising glutamine and lowering glutamate levels, and this was greater in triple-negative than in ER-positive cells. Consistent with the previous study [18], glutamate output was rather higher or not affected in luminal B cells in similar scenarios, and this suggests that cells may be using other pathways or mechanisms to compensate for increased metabolite downstream of glutamine. We observed in *GLS* knockdown and CB-839-treated cells a rise in glutamine levels and a reduction in glutamate. The higher glutamine observed when GLS was knocked down and using the GLS inhibitor in the two luminal B cells meant the mechanisms can block GLS activation, raising glutamine. However, this reduction in glutamine was not sufficient to cause a decrease in cell proliferation. The possible explanation for the difference between siRNA effects and CB-839 insensitivity in luminal cell lines could be that genetic knockdown induces a more profound metabolic disruption than pharmacologic enzymatic inhibition. When GLS is inhibited, residual enzymatic activity together with intact GLS2 function can maintain partial glutamine-derived metabolism through alternative metabolic pathways, allowing some mitochondrial glutamate production and downstream metabolic flux to persist. This residual activity enables adaptive signalling and metabolic compensation. In contrast, genetic knockdown removes the entire protein, abolishing all enzymatic function and more effectively eliminating mitochondrial glutamate generation, leading to greater depletion of intracellular glutamate and an antiproliferative phenotype [27].

In addition, upon knockdown of *GLS2* in the two luminal B cancer cell line models, there was a decrease in cell proliferation. These findings are unexpected, and there are contradictory findings to those of previous researchers’ reports. However, recent studies revealed that the GLS2 inhibitory mechanism on cancer cell proliferation is independent of glutaminase activity [28,29]. The evidence provided by Suzuki and colleagues on GLS2 being a p53 target gene and p53 regulating intracellular glutamine metabolism through GLS2 suggests that it has anti-proliferative effects in tumour cells [17,30], and overexpression of GLS2 reduces aggressive features of tumour cells; hence, the expectation is to have the opposite function upon knockdown. However, a different study reported that *GLS2* knockdown impacts cell proliferation and is needed for glutamine-mediated TCA cycle anaplerosis in ER-positive BC, although the study did not make a distinction between luminal A and luminal B [17].

Together, these findings suggest tumours could have different requirements of GLS2 in glutaminolysis. GLS2 also has proliferative effects in other cancers, suggesting a context-dependent role [24,31]. It is reported that several exogenous and endogenous effectors can affect the activity of glutaminase; hence, a differential functional regulation of distinct isoenzymes and a selective expression of these may result in cancer cells performing glutaminolysis under different conditions.

A similar finding was observed in the current study, where GLS knockdown reduced cell proliferation, consistent with previous findings [18,19]. Altogether, this may suggest co-expression of GLS and GLS2 in luminal BC might be accountable for the prevalent glutaminase activity. There is a possibility that GLS becomes active when GLS2 is knocked down, but this needs further validation, including investigation of mechanisms such as post-translational modification, which are critical for modulating enzyme activity and metabolic adaptation. Furthermore, tumours might overcome GLS2 overexpression by adapting and overexpressing GLS, or we speculate that the function of GLS2 in luminal B cells is negligible. Mechanistic studies are highly warranted to understand the underlying molecular mechanisms.

Even though knockdown of *GLS2* inhibited cell growth and increased apoptosis in luminal B tumours, it did not impact cell-cycle distribution. One of the functions of GLS2 is to lower ROS levels in tumour cells and protect them from oxidative stress-induced cell death. A downregulation of this isoform might have an opposite effect. In addition, the levels of glutamine and glutamate metabolites remained unchanged after knockdown. One of the roles of GLS2 highlighted in hepatocellular carcinoma is the reduction in ROS levels through increasing glutamate levels. GLS2 is critical for glutamine-mediated anaplerosis in luminal BC cells [17]. GLS2 might not be the major driver in the physiological behaviour in this BC subtype and glutamine–glutamate downstream pathway, and cells might be using other metabolic pathways to exert this outcome. Tumour cells expressing either GLS or GLS2 can drive glutaminolysis under diverse metabolic conditions and various substrates, activators, and inhibitors.

In addition, GLS2 is regulated by genes such as p53. Targets and regulators of GLS2, such as p53 or TAP53, should be explored to confirm if GLS2 is upregulated or downregulated. Whilst the current study showed *GLS2* knockdown having very limited effect in luminal B tumours. We acknowledge that there are several limitations in this study, and further research to study the consequences of *GLS2* inhibition or knockdown is required. To further understand the regulation of GLS2 in luminal BC, the mechanisms underlying its gene expression, such as hypermethylation and other factors that may contribute to the expression pattern of GLS2 in BC, should be investigated. Furthermore, to assess post-translational modification of GLS2 to determine the effect of *GLS2* knockdown on cell-cycle progression. There is a need to explore targets and regulators of GLS2, such as *p53*, to confirm if GLS2 is upregulated or downregulated.

The present study shows a distinction between gene expression patterns between *GLS* and *GLS2* in luminal BC. The study also used two cell lines representing HER2-negative luminal B BC. It is important to separate HER2 overexpression from the luminal B cell lines, as it is associated with ER down-regulation. C-Myc regulates GLS, and our current study highlights the potential oncogenic role of glutaminase in BC.

Additionally, simultaneous *GLS*/GLS2 knockdown and assessment of cell growth and other functional studies in luminal B BC are recommended in future studies. In addition to exploring whether there is any synergistic effect from co-targeting both GLS isoforms in luminal BC cell lines and assessing combinations of CB-839 with other drugs to achieve marked therapeutic benefit targeting GLS. Based on our findings, luminal B BC cells are sensitive to *GLS* knockdown and exhibit moderate dependency on GLS. Luminal BC cells are not highly GLS dependent compared to triple-negative BC cells.

## 4. Materials and Methods

### 4.1. Cell Lines and Cell Culture

A panel of human BC cell lines representing different molecular subtypes based on IHC profile, ZR-75-1 and MBA-MB-175VII cells, characterised as luminal B, MCF7 and T47D (luminal A), and MDA-MB-231 (triple-negative BC) [32], were obtained from the American Type Culture Collection (ATCC) (Rockville, MD, USA). ZR-75-1 cells were cultured in Roswell Park Memorial Institute (RPMI) 1640 medium (Sigma-Aldrich, Poole, Dorset, UK) plus 10% heat-inactivated foetal bovine serum (FBS), while MDA-MB-175VII cells were cultured in DMEM/F-12 (Sigma-Aldrich, Poole, Dorset, UK) supplemented with 10% (*v*/*v*) heat-inactivated foetal bovine serum (Sigma-Aldrich, Poole, Dorset UK). Cell lines were incubated at 37 °C and humidified in 5% CO_2_ conditions. Cells were grown and maintained in culture media until they were 80–90% confluent. Three independent experiments were carried out for each assay below.

### 4.2. Transient Knockdown of GLS and GLS2 in Luminal BC Cells

Cells were transfected using the forward transfection method using small interfering RNA (siRNAs) transfection. Pre-validated silencer select siRNA constructs designed for *GLS* and *GLS2* (ThermoFisher Scientific, Vilnius, Lithuania); *GLS*: 5′-UUGUAUAGAACAGCAAAUCtt-3′ and *GLS2:* 5′-UUUGUUACAGUCCAUCUUGat-3′ were used. A total of 250,000 cells/mL were seeded in T25 flasks, and cells were transfected with a 10 nM concentration of siRNAs using Lipofectamine RNAiMAX reagent (13778150, ThermoFisher Scientific, Vilnius, Lithuania) alongside the negative, scrambled siRNA, which was a non-specific scrambled siRNA (Silencer^®^ Select siRNA 4390843, ThermoFisher Scientific, Oxford, Oxfordshire, UK), following the manufacturer’s protocol. After 24 h incubation, the transfection medium (Opti-MEM; Gibco; ThermoFisher Scientific, Vilnius, Lithuania) was removed, and the cells were maintained in normal growth medium. To determine the maximum efficiency knockdown, for each siRNA, a Western blot was performed in cell lysates prepared from transfected and untransfected (control) cells, and protein was quantified. To assess the transfection efficiency and for subsequent functional assays—cell apoptosis, cell cycle, and Glutamine/Glutamate-Glo™ assays (Promega, Madison, WI, USA) were also performed, with the last one measuring relative levels of glutamine and glutamate metabolites (lysates).

### 4.3. Cell Inhibition Using GLS CB-839 Inhibitor

To assess the effect of GLS inhibition in BC cell lines and to ensure the findings of CB-839 inhibition are not limited only to a single cell line, MCF-7, ZR-75-1, MDA-MB-175VII, and MDA-MB-231 cells were exposed to the CB-839 inhibitor (gift from Calithera Biosciences Inc., San Francisco, CA, USA). Briefly, 1000 cells were seeded per well in triplicate in 96-well flat opaque microplates in RPMI-1640 media containing 2 nM glutamine (Corning, Tewksbury, MA, USA) for 24 h. Based on the previous studies using the same inhibitor [18] cells were maintained in the same concentration of glutamine and the same condition of media prior to treatment. To yield consistent results, on the day of compound addition, fresh media was prepared to use for the compound addition. Based on the literature, cells were treated with serial dilutions of CB-839 inhibitor from 0.15 nM to 1000 nM and incubated at 37 °C for 72 h [18]. After 72 h of treatment with the inhibitor, cell viability was measured at 0 h and after 72 h time points, and treated cells were compared with those in the vehicle solvent (DMSO). A total of 100 μL CellTiter-GLO reagent (Promega, Madison, WI, USA) was added to each well and incubated for 10 min in the dark. Absorbance was measured using the Envision Multi-Label Reader (Perkin Elmer, Waltham, MA, USA) at 560 nm. After incubation with the CB-839 inhibitor, glutamine/glutamate production was also measured compared with controls.

### 4.4. Western Blot

Transfected cells were harvested and lysed in mammalian cell lysis buffer, and Western blot analysis was performed as previously described [15]. Membranes were incubated with anti-GLS and anti-GLS2 primary antibodies at optimised dilutions as outlined in [15], with anti-GLS applied overnight at 4 °C and anti-GLS2 for 1 h at room temperature. Anti-β-actin primary antibody (1:5000 Sigma, Life Sciences, Poole, Dorset, UK) was used as an internal reference. This was followed by incubation with donkey anti-mouse and anti-rabbit fluorescent secondary peroxidase-conjugated antibodies (1:15000 IRDye 680RD and IRDye 800CW, LI-COR Biosciences, Lincoln, NE, USA) for 1 h at room temperature. Bands were visualised using a LI-COR Odyssey Fc system with Image Studio 4.0 at 700 nm and 800 nm. Distinct bands were detected at the expected molecular weights, 72 kDa and 65 kDa corresponding to GLS isoforms, and 65 kDa and 31 kDa corresponding to GLS2 isoforms.

The quantification of protein levels in each cell line was measured against normalised β-actin protein from the same membrane. Protein quantification was conducted using Image Studio software version 4.0 (LI-COR Biosciences, Lincoln, NE, USA). Briefly, band intensities were measured by adjusting the boundaries of each protein band and defining the outline of each lane on the blot. Background signal was then quantified and subtracted from the target protein and loading control signals within each lane. The resulting values were subsequently normalised to the internal loading control.

### 4.5. Cell Proliferation

Dimethylthiazol-carboxymethoxyphenyl-sulfophenyl-tetrazolium (MTS) cell viability assay was conducted to determine the effect of *GLS* and *GLS2* knockdown on cell growth in transfected cells (Cell-Titer 96^®^ Aqueous One Solution, G3580 Promega, Madison, WI, USA). At 48 h after transfection, ZR-75-1 and MBA-MB-175VII transfected cells with *GLS* or *GLS2* siRNA and scrambled negative control cells were re-seeded in triplicate at 1000 cells/well in a 96-well plate. On the day of analysis for each time point, 20 μL of the proliferation MTS reagent (Promega, Madison, WI, USA) was added and incubated for 1 h at 37 °C in humidified conditions. The absorbance was measured at 450 nm using a microplate spectrophotometer reader (VersaMax™ Tunable Microplate Reader, Molecular Devices, San Jose, CA, USA). The cell proliferation percentage was calculated relative to the control cells.

### 4.6. Cell Apoptosis and Cell Cycle

After 72 h of knockdown induction with siRNA specific for *GLS* or *GLS2*, 1.5 × 10^5^ transfected and untransfected control cells were harvested in triplicate. Cells were gently trypsinized, washed with 1X PBS, followed by resuspension in 500 μL of 1X Annexin V binding buffer (ab14084, Abcam, Cambridge, Cambridgeshire, UK). Apoptosis analysis was performed by double-staining cells with 0.5 µL Annexin V-FITC (ab14082, Abcam, UK) and 0.5 µL propidium iodide (PI), followed by incubation for 5 min at room temperature in the dark.

For cell-cycle assessment, *GLS* or *GLS2* siRNA-transfected cells and untransfected controls were fixed overnight at 4 °C in 66% cold ethanol. Cell cycle distribution was determined using a PI flow cytometry kit for cell-cycle analysis (ab139418, Abcam, Cambridge, Cambridgeshire, UK) following the manufacturer’s instructions. Briefly, fixed cells were equilibrated at room temperature, gently mixed, and centrifuged at 500× *g* for 5 min. The supernatant was discarded, and cells were resuspended in 1mL PBS and centrifuged again. The resulting pellet was resuspended in a 200 μL mixture of 1X PI + RNase staining solution, ensuring complete dispersion of cells. Samples were covered with foil and incubated at 37 °C in the dark for 25 min. After incubation, tubes were placed on ice, and cells were gently mixed before acquisition. Three independent experiments were performed. Samples were analysed using a MACSQuant^®^ analyser flow cytometer (Miltenyi Biotec, Bergisch, North Rhine-Westphalia, Germany), and data were processed with FlowJo software (version 14.0.0.0., BD Life Sciences, San Jose, CA, USA).

### 4.7. Glutamine/Glutamate-Glo Assay

The Glutamine/Glutamate-Glo™ assay was performed to determine levels of glutamine and glutamate in media following *GLS* knockdown and in untransfected cells. A total of 1000 transfected and untransfected cells were collected and seeded in triplicate in 96-well plates for each time point measurement for glutamine and glutamate. Three independent experiments were performed.

### 4.8. Statistical Analysis

Statistical analysis was performed with GraphPad Prism Version 8.0 (GraphPad Software Inc., La Jolla, CA, USA). The statistical difference was performed by a two-tailed Student’s *t*-test, one-way analysis of variance (ANOVA), or two-way ANOVA test. The post hoc Tukey correction test was applied following ANOVA, where applicable. Data are presented as mean ± SEM from at least three independent experiments, and *p* < 0.05 was considered statistically significant.

## 5. Conclusions

These findings support a functional role for GLS in luminal B breast cancer while suggesting limited pharmacologic sensitivity to CB-839 under standard culture conditions. Future studies are needed to understand the metabolic consequences of targeting glutaminase in luminal BC.

## Data Availability

The data that has been used in this work is available on reasonable request.
